# Improved Solubility and Activity of Natural Product in Nanohydrogel

**DOI:** 10.3390/ph16121701

**Published:** 2023-12-08

**Authors:** Uce Lestari, Muhaimin Muhaimin, Anis Yohana Chaerunisaa, Wawan Sujarwo

**Affiliations:** 1 Doctoral Program, Faculty of Pharmacy, Universitas Padjadjaran, Sumedang 45363, Indonesia; 2 Department of Pharmacy, Faculty of Medicine and Health Sciences, Universitas Jambi, Jambi 36361, Indonesia; 3 Department of Biological Pharmacy, Faculty of Pharmacy, Universitas Padjadjaran, Sumedang 45363, Indonesia; 4 Center of Herbal Study, Universitas Padjadjaran, Sumedang 45363, Indonesia; 5 Department of Pharmaceutics and Pharmaceutical Technology, Faculty of Pharmacy, Universitas Padjadjaran, Sumedang 45363, Indonesia; 6 Ethnobotany Research Group, Research Center for Ecology and Ethnobiology, National Research and Innovation Agency (BRIN), Cibinong, Bogor 16911, Indonesia

**Keywords:** natural products, drug delivery, nanohydrogel, pharmacological activity, solubility

## Abstract

With the development of technology, natural material components are widely used in various fields of science. Natural product components in phytochemical compounds are secondary metabolites produced by plants; they have been shown to have many pharmacological activities. Phytochemical compounds obtained from plants have an important role in herbal medicine. Herbal medicine is safer and cheaper than synthetic medicine. However, herbal medicines have weaknesses, such as low solubility, less stability, low bioavailability, and experiencing physical and chemical degradation, reducing their pharmacological activity. Recent herbal nano-delivery developments are mostly plant-based. A nanotechnology-based system was developed to deliver herbal therapies with better bioavailability, namely the nanohydrogel system. Nanohydrogel is a delivery system that can overcome the disadvantages of using herbal compounds because it can increase solubility, increase pharmacological activity and bioavailability, reduce toxicity, slow delivery, increase stability, improve biodistribution, and prevent physical or chemical degradation. This review article aimed to provide an overview of recent advances in developing nanohydrogel formulations derived from natural ingredients to increase solubility and pharmacological activity, as well as a summary of the challenges faced by delivery systems based on nanohydrogel derived from natural materials. A total of 25 phytochemicals derived from natural products that have been developed into nanohydrogel were proven to increase the activity and solubility of these chemical compounds.

## 1. Introduction

Natural products are the main focus of further research in various fields of science in the world [1,2]. The use of natural products is currently the main choice that can be trusted and has been used for generations in traditional medicine throughout the wider community. It has benefits in treatment or therapy, especially in the use of efficacious active substances derived from components of natural ingredients [3,4]. This is based on ethnopharmacological studies that state that *Cymbopogon jwarancusa* (Jones) Schult. (Family: Poacea/Gramineae) is widely used to treat various diseases empirically in traditional medicine because it has antioxidant, antiallergic, antiparasitic, analgesic, antipyretic, and anticancer activity [5]. Treatments that are carried out using natural ingredients around us are expected to be able to provide extraordinary benefits and impacts by producing fewer side effects than the use of synthetic drugs [6]. Currently, the use of medicines based on natural products has become widely popular among the public, whether in the form of traditional medicine or phytopharmaceutical medicines and standardized herbal medicines. Based on research by Ouoba, K., 2023, it is stated that in practice, the use of phytopharmaceuticals from the collection of wild medicinal plants is the main source for obtaining raw materials (51.5%), which are generally leaves (32.3%) [7]. These raw materials are usually dried in the sun (43.9%) and mostly packaged in plastic bags (37.2%). These medicinal plants come from 60 plant species belonging to 33 plant families. Fabaceae is the most represented family (18.7%), and *Khaya senegalensis* Juss. (Meliaceae) is the most cited plant species (5.2%) [7].

The greater the use of plant parts in herbal medicine by the wider community, the higher the researchers’ curiosity to conduct further investigations about the components of natural ingredients in natural product preparations [8,9]. Natural product components in phytochemical compounds or known as bioactive compounds are secondary metabolites such as alkaloids, flavonoids, steroids, saponins, terpenoids, and tannins. These are produced by plants; they have been shown to have many pharmacological activities [10,11]. *Amaranthus* L. leaves are consumed as a vegetable, are a rich source of secondary metabolites, such as hydroxycinnamic glucaric acid, isocitric acid, caffeoylglucaric acid, and have very strong antioxidant activity [12]. Natural ingredients are used in herbal medicine and therapy, as well as the field of cosmetics based on nature, where they can be developed and empowered as a potential source of products [13]. As reported in [14], cosmetics have adopted natural products as the main and additional ingredients for cosmetic formulations, such as the use of *T. sinensis*, *C. long*a, C. *heyneana*, and *C. zedoria* as anti-aging products in the Anak Dalam tribe community, Jambi province [15].

One way to separate the components of natural ingredients is extraction, where extracts produced empirically are used to treat certain diseases because they contain bioactive compounds or phytochemical compounds that can provide pharmacological effects. With technological development, a technique for separating chemical compounds, chromatography, was discovered to isolate active compounds from natural product extracts into pure compounds. These isolates are tested both in vitro and in vivo to determine the pharmacological effects and bioavailability in the body [16]; additionally, pure compounds isolated from plants can be developed into nanohydrogel, which is able to increase the activity of the pure compound compared with using the pure compound itself. This was proven in the phytochemical compound curcumin produced through isolation from *C. longa* plants, where nanohydrogel preparation containing curcumin had an increased IC_50_ value, showing significantly (2.5-fold) higher efficacy in 4T1 cells (IC_50_, 2 vs. 5 μg/mL) and a two-fold higher efficacy in MiaPaCa-2 cells (IC_50_, 9 vs. 18 μg/mL) compared with curcumin [17].

Nearly 40% more compounds derived from natural products have low water solubility and high toxicity [18,19]. Low solubility in water reduces permeability, that is, the ability to penetrate the barrier, and absorption can affect the bioavailability of a natural compound in the body. In addition, the bioavailability of a compound is also strongly influenced by the stability of the compound against gastric and intestinal pH, metabolism by normal microflora in the digestive tract, absorption through the intestinal wall, the active mechanism of the efflux pump, and first-pass metabolism [20]. Pure compounds resulting from separation from natural products through extraction, fractionation, and isolation have lower solubility in water than pure compounds trapped in nanohydrogel. This form of nanohydrogel is very helpful in overcoming the problem of solubility of pure compounds from natural ingredients. According to research by Li, J et al., 2023, a soy protein isolate with a particle size of 195 nm showed a significant increase in solubility and an increase in affinity and bioavailability of 84.31% when in nanoparticle form [21].

Moreover, using natural or traditional plants as active ingredients in drug formulations still has many limitations, such as poor bioavailability–solubility, gastric pH instability, pre-systemic metabolism in the liver, as well as solubility and absorption problems in the body, which can lead to drug levels below therapeutic plasma concentrations, thereby causing no therapeutic effect from the use of these herbal medicines as drugs or cosmetics [21]. Cosmetics in the form of nanohydrogel can increase the bioavailability and solubility of the pure compounds.

Certain natural product compounds face challenges regarding their bioavailability within the body. For instance, curcumin exhibits very low solubility and undergoes high first-pass metabolism [22]. Quercetin is easily degraded by stomach acid or digestive enzymes, reducing its effectiveness [23]. Emodin is hampered by low percutaneous permeability, affecting its absorption. Silymarin encounters issues due to its low solubility [24], and approximately 80% is excreted through the bile system after undergoing processes like glucuronidation and sulfation [25]. Lastly, naringenin is highly susceptible to degradation when exposed to light, heat, oxygen, or stomach acid [26]. Therefore, it is essential to develop formulas to increase the bioavailability of natural product compounds [27]. A noteworthy advancement in drug and cosmetic delivery systems that addresses the challenge of utilizing pure compounds derived from natural ingredients is the nanohydrogel dosage form. Nanohydrogel comes from Greek and consists of three syllables: “*nano*”, “*hydro*”, and “*gel*”, where “*nano*” means small, “*hydro*” means water, and “*gel*” means to absorb. Therefore, nanohydrogel means a polymer network that can absorb large volumes of water with tiny particle sizes (nanometer) [28].

*Cuminum cyminum* L. is an herbaceous plant of the Umbelliferae family, which is distilled into the essential oil known as caraway oil and is generally used as a flavoring for traditional food. In addition, cumin oil has pharmacological activity as an antifungal and antibacterial medicine. Caraway oil formulated into a nanohydrogel system has increased antifungal activity against *A. terreus* and *A. flavus* at 650 ppm and 350 ppm, respectively. Cumin oil increases its solubility in water if it is encapsulated by nanogels [29]. In addition, nanogel containing apigenin was much more effective in suppressing K562 cell growth than apigenin. Increased solubility of apigenin and increased accumulation in tumors is one of the advantages of the nanohydrogel delivery system [30]. The research findings above show that nanohydrogel preparations help increase pharmacological activity and solubility.

Based on the findings above, the chemical compounds of natural ingredients in a single form have low solubility or activity compared to those in a nanohydrogel system. Nanohydrogel is a more modern drug delivery system that controls drug release to improve pharmacological activity. Nanohydrogel carrier systems derived from natural materials can deliver drugs at an increased rate according to the body’s needs during the treatment period and deliver active compounds from the preparation to the site of drug action quickly [31,32]. These findings show that when using a single pure compound, the solubility is worse in water than in the nanohydrogel dosage form, which is very soluble in water.

The review article about increasing the activity and solubility of natural products in nanohydrogel preparations has significant benefits in developing medicines and medical applications. Using nanohydrogel technology can increase the efficiency of delivering various natural products, such as medicinal plant extracts, natural antibiotics, or other bioactive compounds, into the human body. It allows lower doses achievement of the desired therapeutic effect, reduces the possibility of side effects, and increases drug availability. In addition, nanohydrogels can also increase the solubility of less soluble natural products in water, opening the door to broader use of natural products in pharmaceutical formulations and increasing the clinical benefits that can be obtained from this abundant natural resource.

Nanohydrogel application in herbal products has several advantages for natural compounds, for example, increasing solubility, bioavailability, reducing toxicity, increasing pharmacological activity, slowing delivery, protecting from extreme pH in the stomach, increasing stability, improving biodistribution, and preventing physical or chemical degradation [32]. Nanohydrogel can be used to determine the increase in bioavailability of bioactive and determine biodistribution in specific tissues so that their toxicokinetic profile is known. Preparations based on Nanohydrogel technology can be an alternative to manufacturing natural products. From these formulations, it is hoped that the bioavailability of natural products in the body improves to provide better therapeutic effects. This review article provides an overview of the latest advances in the development of nanohydrogel formulations from natural ingredients that increase the solubility and activity of these natural ingredients. A total of 25 phytochemicals derived from natural products developed into nanohydrogel dosage forms can increase the activity and solubility of these compounds.

The aim of this review article is to examine the mechanism of nanohydrogel as a drug delivery system which can increase the activity and solubility of phytochemical compounds, as well as the advantages of the nanohydrogel delivery system compared to other delivery systems, such as liposomes, phytosomes, micelles, and nanotubes.

## 2. Methods

**Figure 1:**
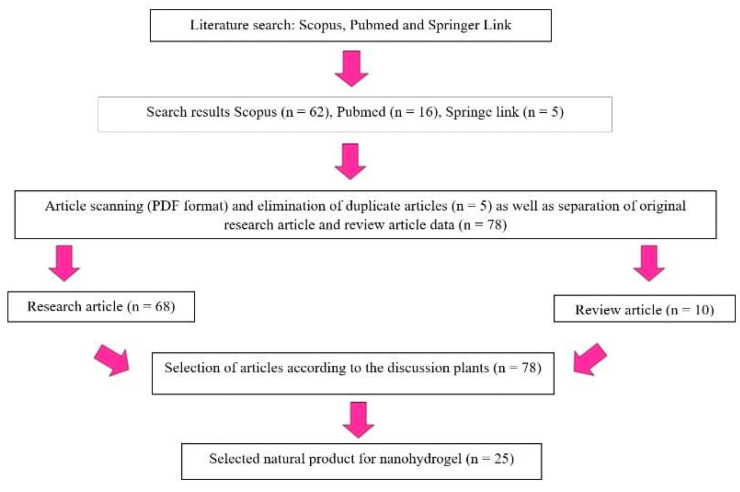
Flow diagram of the study.

The review was conducted in May 2023 and involved several steps. Firstly, a literature search with specific eligibility criteria and keywords was performed using Scopus, PubMed, and Springer Link. The collected articles were then converted into a PDF format. Duplicate articles were eliminated. After that, they were categorized into research articles and review articles. Next, relevant articles were selected based on the topic of interest. Finally, the chosen articles were included in the review. The research flow can be seen in Figure 1 below.

A total of 25 bioactive compounds derived from natural products were developed into a nanohydrogel delivery system, effectively addressing issues related to pharmacological activity and solubility. When these compounds are transformed into a nanohydrogel dosage form, it is evident that their activity increases. In addition, their solubility is enhanced compared to pure bioactive compounds. The articles studied are research articles that use natural ingredients and formulate them into nanohydrogel preparations. Their activity is tested either as a single compound or trapped in a nanohydrogel. In addition, research articles include solubility tests for individual compounds and their nanohydrogel forms, enabling the authors to compare the enhancement in activity and solubility before and after their transformation into nanohydrogel preparations.

## 3. Nanohydrogel

Nanohydrogel is a combination of hydrogel and nanoparticulate systems. Like hydrogels, nanohydrogel can protect drugs and control drug release through biodegradable bonds into polymer networks. Nanohydrogel is a three-dimensional network that holds water inside [32]. Nanogel is a more modern drug delivery system that controls drug release to increase solubility and pharmacological activity. According to research by Gadhave et al. (2021), nanoformulation developed using gellan gum and Carbopol 974P as gel-forming agents can increase anticancer activity against cells and increase drug solubility and permeability [33]. Nanohydrogel containing curcumin with a size of 1–100 nm increases curcumin’s solubility significantly, which is difficult to dissolve more than 100 times, and enhances its anticancer activity against several cancer cell lines [34]. With a tiny particle size, the nanohydrogel dosage form can quickly penetrate semipermeable tissue to provide pharmacological activity. According to research by Saini et al. (2022), nanohydrogel is the most preferred way of preparation for nanoparticle drug delivery because of its intrinsic properties with a polymer system in the form of chitosan with polymer properties of biodegradability, biocompatibility, and non-toxicity [35]. Apart from that, it has attractive properties in gel formation; therefore, it is a suitable carrier for the formulation of nanohydrogel preparations. For this reason, the current sophisticated delivery system for herbal active ingredients is auspicious with the use of chitosan nanoparticles in increasing pharmaceutical efficiency [35].

In the era of nanotechnology, the synthesis of nanoparticles for advanced applications has developed rapidly. Drug therapy and its effective functionalization using nanotechnology are highly productive and selective. Polymer nanomaterials play a significant role and influence this process. Polymer nanomaterials are widely used nowadays due to their low cytotoxicity, strong functionalization, and practical approach to in vitro and in vivo therapy [36]. The use of nanohydrogels with polymers has developed significantly in the last few decades by minimizing the side effects that occur and providing a constructive strategy that can address essential goals in nano-therapy systems [36].

Nanohydrogel formulation targets drug and cosmetic delivery systems that can increase solubility and bioavailability, reduce toxicity, enhance pharmacological activity, slow delivery, increase stability, improve biodistribution, and prevent physical or chemical degradation. Nanohydrogel is one of the best delivery systems currently. Nanohydrogel derived from natural products is the best combination for developing drug and cosmetic products [37,38,39]. Today, the cosmetic delivery system in the form of nanohydrogel derived from natural ingredients is a prevalent choice for the public because it quickly provides activities such as anti-aging and has minimal side effects. It is confirmed by research conducted by Parhi et al. (2022), which explains that drug delivery systems (DDS) with the target of topical drug administration have advantages compared to oral and parenteral routes, including avoiding first-pass metabolism, preventing drug degradation, administering controlled medication, and minimizing pain when administering it. DDSs with a suitable polymer are the main requirement in nanohydrogel formulation [40]. Based on current trends, natural polymers are more popular than synthetic polymers because natural polymers are non-toxic, biodegradable, biocompatible, low-cost, sustainable, and a renewable resource. For example, polysaccharides consisting of monosaccharide chains held together by glycosidic bonds have successfully enhanced drug delivery into and across the skin in nanohydrogel formulations. Furthermore, the saccharide polymers used include cellulose, chitosan, and their semisynthetic derivatives, such as alginate, pectin, HPMC, PLGA, carrageenan, and others [40,41].

One of the main techniques used to synthesize nanohydrogel is the ionic gelation method. Nanohydrogels are synthesized through chemical cross-linking formed through covalent bonds or physical cross-linking through noncovalent bonds. Based on research by Kaur et al. (2022), ionic gelation is the primary technique for forming milk protein nanohydrogel [36]. The milk protein-based nanohydrogel-forming system can improve various functions, such as food nutrition and health, food engineering and processing, and food safety [41]. Therefore, milk protein nanohydrogels have many applications in the food industry because denaturation, aggregation, and gelation of proteins have special meaning in making new nanostructures, such as nanohydrogels, in various scientific fields of application [41,42].

**Figure 2:**
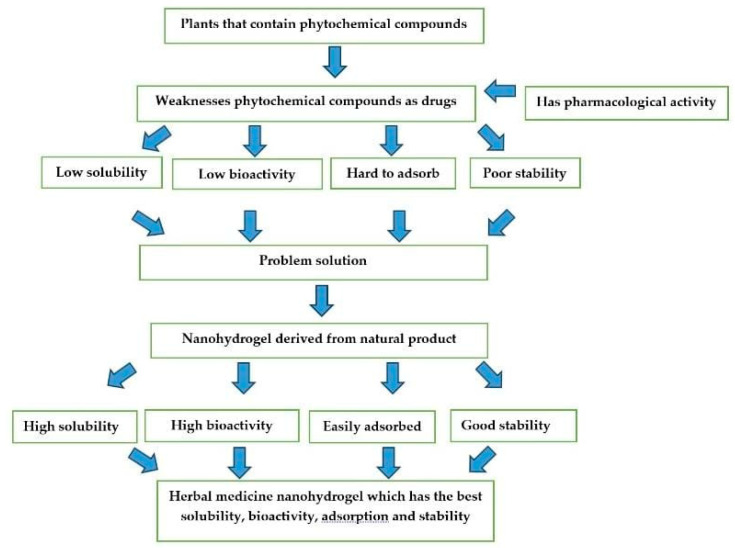
Flow of ways to overcome the weaknesses of phytochemical compounds in medicines.

However, using natural ingredients as active ingredients has many limitations, including poor solubility and bioavailability, difficulty being absorbed, and instability as a drug [42,43]. Therefore, the drug and cosmetic delivery system, in the form of nanohydrogels, can overcome all these problems. Ways to overcome the weaknesses of phytochemical compounds in drugs can be seen in Figure 2 below.

## 4. Nanohydrogel-Based Drug Delivery System for Natural Product Compounds

Natural ingredients are beneficial for medical therapy in humans. It is because they contain phytochemical compounds that can be natural therapies, such as herbal plants that grow in the local environment. Some of these herbal plants can inhibit cancer growth. Phytochemical compounds isolated from herbal plants can be used in nanotechnology medicine [44]. Currently, nanoparticle delivery systems are used for facial skin care. However, many cancer treatments have been applied using pure compounds from natural ingredients in the form of nanoparticles because the activity inhibits the growth of cancer cells more quickly than when using a single pure compound.

Natural products are the best source of bioactive chemicals used in the medical and pharmaceutical fields because they can succeed in therapy, offer outstanding results in both in vitro and in vivo responses, have high-loading amounts of drugs, and have good cell absorption. One of the most promising and significant delivery agents for drug delivery throughout the body is nanohydrogel [44].

In preparing products based on natural ingredients, paying attention to problems that often occur, such as the solubility and bioavailability of the bioactive compounds contained therein, is essential. One way to overcome this problem is to take advantage of developments in pharmaceutical technology, namely the Novel Drug Delivery System (NDDS). One of the carrier systems included in NDDS is a polymer-based carrier system that modifies the solubility of flavonoid compounds, such as chitosan nanoparticles, which can potentially increase the bioactivity of natural or phytochemical compounds in nanohydrogel preparations [43,44,45]. The preparation of nanohydrogel depends on the polymer used. The most widely used polymer is chitosan because it has many advantages, such as being non-toxic when mixed with other polymers, being stable during use, having a high surface area, and being used as a constituent for various drugs or plant extracts [44].

**Figure 3:**
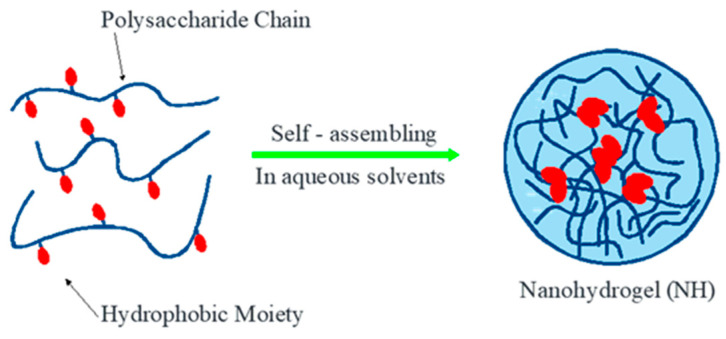
Drug delivery system with a polymer carrier system (nanogel polymer).

Polymers have been used in medicinal and cosmetic products as materials for drug delivery systems. The use of polymers as carriers in nanohydrogel formulations is inert to active ingredients derived from natural materials or phytochemicals. Polymers can form networks to be developed as carrier systems in particle matrices, such as HPMC, PLGA, pectin, alginate, and chitosan [45]. The following Figure 3 is a picture of a drug delivery system using a polymer carrier system.

Curcumin with a polymer carrier system in a nanohydrogel can increase its stability, sustainable drug release, and solubility in water [46]. Curcumin has pharmacological activity as an anticancer agent. However, direct administration of oral curcumin in clinical trials results in limited therapy. Currently, curcumin derivatives in the form of nanoparticle formulations have advantages over drug protection, sustained drug release kinetics, and increased activity against cancer cells [47]. From the research findings above, it can be explained that the curcumin nanohydrogel form can increase activity in fighting cancer cells compared to using a single curcumin compound.

**Figure 4:**
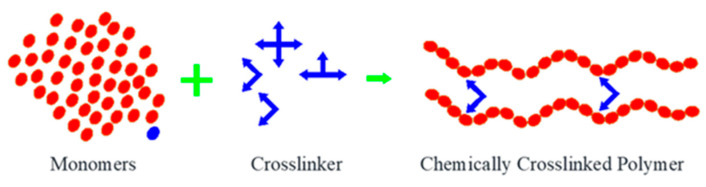
Synthesis of nanohydrogel in oil or water phases.

The synthesis of nanohydrogels is mainly based on natural polymers, such as chitosan and alginate, with the help of cross-linkers like Natrium Tripolifosfat (NaTPP) for the formation of chemical crosslinks [48]. The process of forming nanohydrogel can be described as in Figure 4 below.

Nanohydrogels are formed through a chemical process known as crosslinking, where cross-linkers link polymer molecules together, creating a three-dimensional network that holds water within [48]. This process usually begins with making a polymer solution containing a cross-linker. After that, this solution can be exposed to various methods, such as radiation, heat, or certain chemicals, which initiate the crosslinking reaction. With this reaction, the polymer molecules agglomerate and form a nanohydrogel network with nano sizes that can absorb and release water or other substances very efficiently. This mechanism has been applied in biomedical and pharmaceutical applications, such as drug-targeted delivery, wound healing, and other nanomedical delivery systems [47,48,49].

Nanohydrogels have a smaller surface-to-volume ratio because delivery through cell membranes is more efficient than delivery systems such as liposomes, micelles, nanoparticles, and nanotubes. Nanohydrogel has better absorption capacity than liposomes, phytosomes, and micelles. Therefore, it can deliver drugs or active substances to target tissues more efficiently. In addition, nanohydrogels can be customized to control the gradual release of active substances, providing advantages in long-term therapy. Furthermore, nanohydrogels can overcome several obstacles often faced by liposomes, phytosomes, and micelles, such as better stability in various environmental conditions and the ability to transport various active substances [48,49,50].

Nanohydrogel has the advantage of being hydrophilic and flexible to increase water absorption [48]. In addition, the parenteral route nanohydrogel has good conductivity as it can move in the fine capillaries because of its small size. Thus, the nanohydrogel formulation is a better delivery system for natural product compounds or phytochemical compounds than hydrogel systems [49,50,51].

This study revealed that topical nanohydrogels have higher activity on the skin than hydrogel formulations. Besides that, they can increase the local action and stability of the formula [52]. These findings confirm that protein encapsulation in lipid vesicles in the matrix space in a gel network system enhances the local action of drugs on the skin, reduces their systemic toxicity, and increases drug retention in skin epithelial cells. Thus, 1% (*w*/*w*) protein nanohydrogel preparations have therapeutic potential for managing precancerous skin lesions [50,51,52]. The nanohydrogel dosage form is beneficial not only for oral but also for topical use. Based on the research results above, the nanohydrogel form can accelerate the recovery of skin lesions in cancer patients [53].

Curcumin is a natural compound with anticancer and anti-inflammatory activity. However, the therapeutic efficacy of curcumin has been challenged in clinical trials, primarily due to its low bioavailability, rapid metabolism, and elimination. This study devised a nanoparticle form of curcumin to stabilize and substantially increase its cellular permeability and anticancer activity in standard oral administration. Curcumin conjugated as an ester to cholesteryl-hyaluronic acid nanohydrogels can target delivery to drug-resistant cancer cells expressing CD44 [46,53,54]. From the research above, it is clear that curcumin nanohydrogel can increase cell permeability and pharmacological activity when used orally.

Herbal essential oils, such as *Cuminum cyminum*, are natural antifungal agents with many volatile compounds. This study used encapsulation with chitosan caffeic acid nanohydrogels to increase the antimicrobial activity and stability of *C. cyminum* essential oil against *A. flavus* [52,53,54]. From the research above, it is evident that evaporated oil derived from natural ingredients will be stable in storage if it is transformed into evaporated oil nanohydrogel. Furthermore, pharmacological activity increases in inhibiting the growth of microorganisms.

Curcumin nanohydrogel exhibits excellent solubility and sustained drug release under physiological conditions. It is well tolerated and results in up to 13-fold tumor suppression, thereby making this nanohydrogel drug a potential candidate for cancer prevention and treatment [46,55]. According to the research above, the drug delivery system is based on nanohydrogel. The following Table 1 is list of nanohydrogel with several bioactive compounds.

### 4.1. Nanohydrogel Natural Ingredients Increase the Activity of Compounds

Ganoderma fruit extract with the active compound saccachitin can heal skin wounds by inducing cell proliferation. The study investigated nanogels formed from micronized sacchachitin (mSC) to treat corneal burns potentially. In vitro cell proliferation studies revealed that at concentrations of 200, 300, and 400 µg/mL, mSC nanogel significantly increased the proliferation of Statens Seruminstitut Rabbit Cornea (SIRC) cells after 24 h of incubation and wound closure after 24 h of incubation with the addition of 200 µg/mL mSC nanogels. In animal studies, accelerated mSC nanogels improved corneal wound healing compared to saccachitin extracts [53].

Resin from *B. serrata* contains boswellic acid (BAs). For herbal treatment, *Boswellia serratta* extract sprayed dry on inflammatory disorders inhibits 5-lipoxygenase (5-LO). The form of boswellic acid nanohydrogels (BAs) in ex vivo skin permeation and in vivo anti-inflammatory studies showed a 1.45-fold increase in flux compared to gel and increased drug penetration into the skin [54].

Synthetic compounds often cause skin inflammation, have inadequate penetration, and have unclear clinical efficacy. As a result, preparations with herbal plant components for treating pigmentation disorders and whitening cosmetic agents in previous studies demonstrated the potential of two natural ingredients: salidroside and paeonol. The release of the drug from salidroside and paeonol at a concentration of 0.2 mg by the nanohydrogel system was shown to significantly increase the anti-melanogenesis effect and reduce potential toxicity when used in high doses. The nanohydrogel system as a carrier for delivery has excellent potential in nanomedicine. However, applying these salidroside and paeonol compounds as drugs can be severely hampered by their low stability, poor skin penetration, and potential toxicity at high doses [55]. Changing the form of salidroside and paeonol nanohydrogel can overcome all problems, such as increased stability and good penetration into the skin.

**Figure 5:**
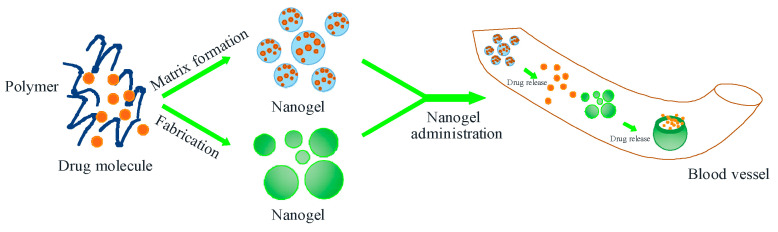
Release of nanohydrogel in the blood.

Curcumin is a natural polyphenol extracted from turmeric with biological activities, including antioxidant, anti-inflammatory, and anticancer activities. However, the therapeutic efficacy of curcumin has been challenged in clinical trials, primarily due to its low bioavailability, rapid metabolism, and elimination. Thus, the researchers devised a nanohydrogel form of curcumin to stabilize and substantially increase its cellular permeability and anticancer activity in standard oral administration. Curcumin conjugated as an ester to cholesteryl–hyaluronic acid nanohydrogels can target delivery to drug-resistant cancer cells expressing CD44. CUR CHA-nanogel treatment resulted in up to 13-fold tumor suppression, making this nanodrug a potential candidate for cancer prevention and treatment [46,53,54,55]. The advantages of this nanohydrogel form are enormous, increasing pharmacological activity by two times and reaching tens of times compared to using a single pure compound. Figure 5 shows the process of releasing nanohydrogel in the blood.

The release of calcitonin from *A. retroflexcus* extract was observed, where the amount of drug release obtained after 72 h was 66.5%, while that of the *A. retroflexcus* extract nanohydrogel was 76%. It was more beneficial than releasing the liver cancer drug from doxorubicin nanohydrogel, which only reached 61.73%. Hence, nanohydrogel from *A. retroflexcus* extract can increase anticancer activity compared to doxorubicin nanohydrogel or *A. retroflexcus* extract. In addition, the drug release for calcitonin from *A. retroflexcus* extract was comparable to that found in other studies, which achieved a 65.1% release of Carum copticum essential oil (CEO) nanohydrogel. In this study, the function of *A. retroflexcus* extract was better than that of Carum copticum essential oil (CEO) [56].

Apigenin is a flavonoid compound in vegetables and is essential to cancer prevention. Apigenin nanohydrogel can suppress the growth of K562 cells depending on the dose and exposure time. This study compared the effect of apigenin nanohydrogel with that of pure apigenin on the K562 cell line derived from CML patients. This study found that the cytotoxic effect of apigenin on K562 cells had an IC_50_ value of 20 mmol observed after 48 h of exposure, while the IC_50_ value for apigenin nanogels was 10 mmol. Therefore, it can be concluded that the toxic effects of apigenin and nanohydrogel increased with increasing concentration and duration of exposure compared to controls. The toxic effect of apigenin nanohydrogel with a stearic–chitosan base is more significant than that of apigenin by twofold [30,53,54,55,56].

Silymarin is extracted from the seeds and fruit of the thistle plant *S. marianum*, the the main biologically active component of which is silibinin. However, the clinical application of silibinin shows some limitations due to its low water solubility, poor penetration into intestinal epithelial cells, high metabolism, and rapid systemic elimination. Nonetheless, nanohydrogel-based drug delivery systems can explore the great potential of phytochemicals to increase the water solubility and bioavailability of BCS class II and IV drugs, improve stability, and modify pharmacological activity [57].

Myristoylated chitosan (MCS) silibinin nanogel was developed for breast cancer therapy. The obtained MCS nanogels are considered suitable drug carriers with high loading capacity to treat cancer cells with lower side effects. The nanoparticle size (MCS) and loading efficiency were around 20 nm and 85–95%, respectively, whereas the MCS nanogels were spherical and homogeneous with a solid surface [57].

Fatty acid methyl esters from sunflower oil can be stabilized as CS-CA nanohydrogels. This study compared the oxidative stability of the O/W emulsions stabilized with CS-CA and Tween 80 nanohydrogels. The O/W emulsions stabilized by CS-SA nanogels had higher oxidative stability than the O/W emulsions stabilized by Tween 80. CS-SA nanohydrogel dissolves at low pH values due to increased electrostatic repulsion, and CS is a cationic polysaccharide with a more positive charge [58]. This form of nanohydrogel is able to increase not only activity and solubility but also stability.

Antioxidants are used to treat aging problems and pathological conditions of the body. Antioxidants are derived from plant extracts from phyto-bioactive compounds, such as curcumin, resveratrol, catechins, and quercetin. Curcumin is one of the most frequently used natural compounds as a potential modulator of the cell damage caused by free radicals. *Curcuma longa* (turmeric) contains curcumin. Specifically, the antioxidant activity of curcumin is determined by the methylene hydrogen and o-methoxy phenolic groups. The therapeutic potential of curcumin has drawbacks, such as its low bioavailability associated with poor solubility, stability, and absorbance in the digestive tract. In the present day, new nanotechnology-based systems are developed for the therapeutic delivery of natural antioxidants with improved bioavailability and, consequently, efficacy in clinical practice. The delivery system is a nanohydrogel with advantages, including superior solubility and stability, extended half-life, better epithelial permeability and bioavailability, improved tissue targeting, and minimized side effects [59].

According to this study, the oral bioavailability of curcumin nanohydrogel poly lactic-co-glycolic acid (PLGA) is 22 times higher than that of free curcumin. In addition, curcumin’s water solubility and distribution are 16-fold higher in the brain than those of free curcumin. Similarly, the oral bioavailability and cerebral distribution of curcumin are significantly enhanced in nanohydrogels with chitosan polymer compared to native curcumin [59].

Green tea extract has potent antioxidants known for their beneficial health effects. One of these compounds, epigallocatechin-3-gallate, has chemotherapeutic properties and can lower blood cholesterol levels. Topical drug delivery is difficult, so nanohydrogels have great potential to overcome this problem due to their reduced particle size and structural properties [38,57,58,59].

*Ammi visnaga* L. is a plant that grows naturally in Europe and is very common in Turkey. It has various pharmacological effects due to the content of γ-pyrone, coumarin, flavonoids, and essential oils. *A. visnaga* flowers contain 22.6 ± 0.06 mg/g khellin. Khellin is the main phytoconstituent obtained from flower and seed extraction of *A. visnaga*, which is effective in the photochemotherapy of skin diseases. Topical delivery of khellin has been enhanced by using nanocarriers, such as nanohydrogel, to achieve optimal efficacy and khellin-related stability for treating several skin diseases, especially psoriasis, eczema, alopecia areata, and vitiligo [60]. The following Table 2 is data of comparison of IC_50_ phytochemical compounds with nanohydrogels. These data were adapted with permission from Eid et al. (2021) and Subhash et al. (2021) [24,34].

The cytotoxicity of CHA-CUR was compared to that of free curcumin in human pancreatic cancer cells MiaPaCa-2 and murine breast cancer 4T1 cells in tumor growth inhibition studies in mouse cancer models. In the thiazolyl blue (MTT) cytotoxicity assay, CHA-CUR showed a significant 2.5-fold higher efficacy in 4T1 cells (IC_50_ = 2 vs. 5 μg/mL) and a 2-fold higher efficacy in MiaPaCa-2 cells (IC_50_ = 9 vs. 18 μg/mL) compared to curcumin [46,61]. It can be concluded that the activity of curcumin nanohydrogel against human pancreatic cancer cells MiaPaCa-2 and murine 4T1 breast cancer cells increased compared to that of free curcumin.

Trypan Blue staining showed the viability of cells treated with apigenin and apigenin nanohydrogel where the cells were counted. The IC_50_ values for apigenin after 24, 48, and 72 h were 50 ± 1.7 µmol/mL, 20 ± 1.66 µmol/mL, and 10 ± 1.48 µmol/mL, respectively. Meanwhile, the IC_50_ values for apigenin nanohydrogels after 24, 48, and 72 h were 20 ± 1.07 µmol/mL, 10 ± 0.53 µmol/mL, and 5 ± 0.51 µmol/mL, respectively. Moreover, the MTT test showed the results of the viability of cells treated with apigenin and apigenin nanohydrogel where cells were counted. The IC_50_ values for apigenin concentrations after 24, 48, and 72 h were 50 ± 1.79 µmol/mL, 20 ± 0.82 µmol/mL, and 10 ± 0.92 µmol/mL, respectively. In contrast, the IC_50_ values for apigenin nanohydrogels after 24, 48, and 72 h were 20 ± 1.07 µmol/mL, 10 ± 1.98 µmol/mL, and 5 ± 0.65 µmol/mL, respectively [30,62]. It can be concluded that the activity of the curcumin nanohydrogel against K562 cells at a small concentration increases compared to that of free curcumin.

Chung et al. found that the toxicity of apigenin to K562 cells depended on dose and time. The cytotoxic effect of apigenin on K562 cells using the MTT test had an IC_50_ value of 16 mmol after 48 h. In comparison, apigenin on K562 cells had an IC_50_ value observed after 48 h of 20 mmol, and the IC_50_ value for apigenin nanohydrogel was 10 mmol [30,62]. It can be concluded that the effect of apigenin nanohydrogel is twofold at the same concentration and time compared to free apigenin.

Ratan et al. stated that the apoptosis levels of using apigenin and apigenin nanohydrogels after 24 h were 30.34% and 38.81%, respectively. Furthermore, apoptosis of K562 cells was induced by apigenin, and no toxic effects were observed on apigenin nanohydrogels on normal blood cells [47,63]. It can be concluded that the level of apoptosis of apigenin nanohydrogel is higher than that of free apigenin.

### 4.2. Nanohydrogel Natural Ingredients Increase the Solubility of Compounds

Ganoderma fruit extract with the active compound saccachitin can heal skin wounds by inducing cell proliferation. This study investigated nanogels formed from micronized sacchachitin (mSC) to potentially treat corneal burns. Ganoderma containing sacchachitin (mSC) is less soluble in water than its nanohydrogel form [53,64].

Resin from *B. serratta* contains boswellic acid (BAs). For herbal treatment, the Boswellia serratta extract sprayed dry on inflammatory disorders inhibits 5-lipoxygenase (5-LO). After determining the solubility of boswellic acids (BAs), it was proven that BA showed the highest solubility in isopropyl myristate (192.71 µ 0.89 mg/2 mL) due to the polarity of the drug. In contrast, its solubility in oil was small/moderate [54,65].

As a result, preparations with herbal plant components for treating pigmentation disorders and whitening cosmetic agents in previous studies demonstrated the potential of two natural ingredients: salidroside and paeonol. The nanohydrogel contains lipids and allows for a much higher solubility of the lipophilic paeonol, in which the drug is quickly loaded to higher amounts. The paeonol can be easily released by drug diffusion or matrix erosion. At the same time, the release of salidroside from the nanohydrogel is only slightly affected by the incorporation of paeonol nanoparticles in the nanohydrogel [55,66].

Curcumin is a natural polyphenol extracted from turmeric with biological activities, including antioxidant, anti-inflammatory, and anticancer activities. CHA-CUR nanohydrogels exhibit excellent solubility and sustained drug release under physiological conditions [46].

The essential oil of *Cuminum cyminum* is a natural antifungal agent consisting of many different volatile compounds. In this study, CS-CA nanogels were synthesized by the aggregation phenomenon through modification of water-soluble CS molecules while CA is hydrophobic. Then, the resulting CS-CA nanogel containing a hydrophilic layer was used to entrap the hydrophobic *C. cyminum* essential oil to achieve oil release into the fungal medium [52,67].

Apigenin (C15H10O5) is a flavonoid compound widely found in vegetables and is essential in cancer prevention. Increased solubility of hydrophobic drugs (apigenin) and increased accumulation of apigenin in tumors are the advantages of the delivery system in the form of nanohydrogels [30,68].

PLGA curcumin nanohydrogel was achieved using a chitosan polymer nanocarrier-based system, resulting in increased solubility and stability of bioactive phytochemicals, increased gastrointestinal absorption, and protection against enzymatic degradation [59].

Green tea extract has potent antioxidants known for their beneficial health effects. The nanohydrogel containing green tea extract had excellent water solubility, and the NG15 nanohydrogel showed the optimal drug content of 99.87% [38,70].

*Ammi visnaga* is a plant that grows naturally in Europe and is very common in Turkey. It has various pharmacological effects due to the content of γ-pyrone, coumarin, flavonoids, and essential oils. Nanohydrogels have a large surface area, increase solubility, and are ideal delivery systems for hydrophobic and hydrophilic drugs. In addition, it is flexible, biocompatible, stable, has a long residence time in the skin, can easily penetrate tissues, has high drug-carrying capacity, significantly reduces drug side effects, and increases its therapeutic efficacy because it is controlled and targeted. In addition, khellin has low solubility in water (0.025% *w*/*v*) and has Log P ≈ 3. Therefore, it is necessary to develop a nanohydrogel-based nanocarrier system in which the properties of drug release and drug penetration into the skin increase so that the concentration of khellin is therapeutic and can reach deep layers [60,71].

## 5. The Challenge of Nanohydrogel Formulations Comes from Natural Ingredients

Since ancient times, herbal medicines, such as turmeric, aloe vera, fennel, clove, eucalyptus, garlic, ginseng, ginger, liquorice, and nutmeg, have been widely used worldwide for local and systemic applications. However, most herbal medicines have limited applications due to their low water solubility, leading to low bioavailability, causing the drugs to be classified as poor candidates for therapeutic applications. Recently, incorporating a new delivery system for herbal medicines in nanohydrogels has shown significant progress in enhancing bioavailability and therapeutic effects [57,72].

Silymarin is extracted from the seeds and fruit of the *S. marianum* plant, which contains flavonolignans, silybin, silydianin, isosilybinin, and silychristin compounds. Among these constituents, the biologically active component is silibinin (silybin). Silibinin has anticancer, antioxidant, anti-inflammatory, hypocholesterolemia, cardioprotective, neuroprotective, and hepatoprotective activities. Silibinin is highly soluble in polar aprotic solvents, such as dimethylsulfoxide (DMSO), acetone, dimethylformamide (DMF), and tetrahydrofuran (THF). However, it is insoluble in non-polar solvents like chloroform and petroleum ether. Silibinin is easily oxidized to 2,3-Dehydrosilybin. Silibinin is considered safe without significant adverse effects [61]. Nanohydrogel-based drug delivery systems can overcome the problems of silymarin therapy by exploring the great potential of phytochemicals to increase the water solubility and bioavailability of Class II and IV BCS drugs, improve stability, and modify pharmacological activity [57,73].

Delivery systems based on nanohydrogels with natural components have many advantages, including increased solubility, increased bioavailability, increased pharmacological activity, increased stability (active ingredients), prevented active ingredients from chemical and physical degradation, and reduced dosage required. Currently, the use of herbal medicines as alternative medicine is widely popular. However, nanohydrogel formulations face challenges in their production processes, such as high production costs, difficulty in processing scale, and lack of data regarding the safety and toxicity of natural components in the form of nanohydrogels [74,75]. Based on that, problems related to nanohydrogel-based drug delivery systems must be considered. Another challenge for nanohydrogels is the stability of these natural materials in the form of nanoparticles. The problem of the ability of nanohydrogels to retain drugs is increasing where nanohydrogels dissolved beforehand before mixing with blood [73,76,77].

The phytochemical compound nanohydrogel system is significantly more expensive to produce, which results in a higher selling price. In European countries, drugs are selected and funded from public sources on a rational selection basis, where high production costs can be a severe constraint. In these countries, high-priced drugs in a nano form have meager chances of reaching the market and patients [74,77,78].

## 6. Conclusions

Natural product components in the form of phytochemical compounds are secondary metabolites produced by plants; they have been shown to have many pharmacological activities. Phytochemical compounds in a single form have low solubility or activity; therefore, to overcome these problems, a new drug delivery system, known as nanohydrogel, was developed. Nanohydrogel is a more modern drug delivery system that controls drug release to improve pharmacological activity. The nanohydrogel carrier system derived from natural ingredients can deliver drugs at an increased rate according to the body’s needs during the treatment period and quickly distribute the active compounds from the preparation to the site of drug action. However, the development of this nanohydrogel-based delivery system still needs further review, especially regarding its safety and toxicity profiles, so that its safety and effectiveness can be ensured for curing various types of diseases.

## Figures and Tables

**Table 1:** Nanohydrogel with bioactive compounds.

No	Bioactive Compounds	Solubility Bioactive Compounds	Nanohydrogel Preparation Method	Dose/Distribution/Toxicity/Loading Efficiency	Improved Activity	Improved Solubility	Nanohydrogel Characteristics	Ref.
1	Apigenin isolate	Apigenin is hydrophobic (difficult to dissolve in water).	Stearate and chitosan polymers	The cytotoxic effect of apigenin on K562 cells had an IC_50_ of 20 mmol, whereas the IC_50_ for apigenin, when delivered with nanohydrogel, was 10 mmol.	Apigenin nanohydrogel can suppress the growth of K562 cells.	Nanohydrogel apigenin is hydrophilic (easily soluble in water).	In materials engineering, apigenin could be used as a thermal stabilizer of starch.	[30]
2	Epigallocatechin-3-gallate from *C. sinensis* extract	Green tea extract exhibits high solubility in water.	Chitosan polymer	The concentration of epigallocatechin-3-gallate used was 1%.	Nanohydrogel enhances chemotherapeutic properties and reduces blood cholesterol levels.	Nanohydrogel with epigallocatechin-3-gallate shows excellent water solubility.	The prepared chitosan nanohydrogel, NG15, demonstrates a % encapsulation efficiency of 99.87%.	[38]
3	Curcumin from *C. domestica* Val. extract	Curcumin has poor water solubility.	Synthesized with modifications using a method where a cholesteryl-amine linker is created through the reaction of cholesteryl chloroformate	Curcumin was tested at 5, 10, 15, and 20 µg/mL concentrations.	A nanodrug containing curcumin enhances stability, cellular permeability, and anticancer activity.	Nanohydrogel with curcumin demonstrates excellent solubility, surpassing the solubility of pure curcumin compounds in water.	Nanohydrogel with curcumin consists of particles measuring 20 nm in size.	[46]
4	Essential Oils from *C. cyminum* extract	Essential oils are hydrophobic (not readily soluble in water).	The aggregation phenomenon	Concentrations of essential oils were 650 and 350 ppm.	Chitosan nanohydrogel essential oils enhance antimicrobial activity.	Nanohydrogel with essential oils is hydrophilic (easily soluble in water).	For the IR spectroscopic analysis, 2 mg of *C. cyminum* (Ci), 2 mg of chitosan (CS), and 100 µL of CS–Ci nanogel are used.	[52]
5	Salidroside and paeonol isolates	Paeonol, a lipophilic compound, has a significantly higher solubility than salidroside.	The nanosphere-gel system	The concentrations of salidroside and paeonol were both 0.2 mg.	The nanosphere-gel system significantly enhances the anti-melanogenesis effect.	Paeonol in the nanosphere-gel dissolves more easily in oil than pure paeonol compounds.	The nanosphere-gel size is 286 nm, with a PDI of 0.397, a zeta potential of −22 mV, and paeonol encapsulation of 65.33%.	[53]
6	Sacchachitin from *G. tsugae* extract	Sacchachitin is sparingly soluble in water.	The ball milling method	The concentration of sacchachitin was 200–400 µg/mL.	Nanohydrogel formed from micronized sacchachitin shows enhanced corneal burn repair compared to Ganoderma fruit extract.	Nanohydrogel sacchachitin is more water-soluble than pure sacchachitin.	Nanohydrogel sacchachitin has particles ranging in size from 10 to 50 μm and possess a negative charge of approximately −22.59 mV.	[54]
7	Boswellic acid from *B. serrata* extract	Boswellic acid exhibits high solubility in isopropyl myristate and low-to-moderate solubility in oil.	Involving a high-speed homogenizer at 6000 RPM for up to 10 min	The concentration of *Boswellia serrata* extract was 133.2 mg.	Nanohydrogel boswellic acid enhances bioavailability as an anti-inflammatory.	Nanohydrogel boswellic acid dissolves more readily in oil than pure boswellic acid.	The particle size of nanohydrogel boswellic acid is 0.22 μm.	[55]
8	Calcitonin from *A. retroflexus* extract	Calcitonin is highly soluble in water.	The nanocomposite is in gel form	The distribution of A. *retroflexus* extract was 80.1% over 7 days.	Nanohydrogel *A. retroflexus* extract enhances anticancer activity.	Nanohydrogel calcitonin is easily soluble in water.	Chitin-poly (L-lactic acid) composite nanohydrogels in PBS achieve an efficiency of 61.73%. Nanohydrogel sizes range from 28.48 to 176.7 nm, with a nanoparticle size of 25.6 nm at pH 4.	[56]
9	Silymarin and silibinin from *S. marianum* extract	Silymarin and silibinin have low solubility in water.	Chitosan polymer	Nanoparticles had a loading efficiency of 85–95%.	Nanohydrogel chitosan, silymarin, and silibinin show enhanced antimicrobial activity.	Nanohydrogel silymarin and silibinin are more water-soluble than pure compounds.	The particle size of nanohydrogel silibinin is approximately 20 nm, with a loading efficiency of 85–95%. Nanohydrogel silibinin is spherical, homogeneous, and has a dense surface.	[57]
10	Fatty acids and methyl esters from sunflower extract	Fatty acids and methyl esters are poorly soluble in water.	Chitosan-stearic acid polymer	The most suitable was the nanohydrogel with chitosan polymer and stearic acid in a 20:1 ratio.	Nanohydrogel *A. retroflexus* extract enhances anticancer activity.	Nanohydrogel methyl esters are soluble at low pH values and more soluble in water than pure methyl esters.	Methyl ester-chitosan-stearate nanogel at pH 8 with a ratio of 0.5:1 has a particle size ranging from 12.0 to 38.1 nm. In comparison, the fatty oil nanogel with a ratio of 20:1 at pH 10 has a particle size ranging from 15.6 to 45.3 nm.	[58]
11	Curcumin from *C. longa* isolate	Curcumin exhibits limited solubility in water.	Chitosan polymer	The concentration of curcumin used ranged from 50 ppm to 200 ppm.	Nanohydrogel curcumin can effectively inhibit the growth of K562 cells.	Nanohydrogel with curcumin demonstrates excellent solubility and is more water-soluble than pure curcumin.	The particle size of nanohydrogel curcumin is 20 nm.	[59]
12	Khellin from *A. visnaga* isolate	Khellin exhibits poor solubility in water.	Chitosan polymer	The concentration of khellin used was 22.6 mg.	This treatment shows promise for skin diseases such as psoriasis, eczema, alopecia areata, and vitiligo.	Nanohydrogel khellin is more water-soluble than pure khellin.	The nanohydrogel khellin is a carrier system with an average diameter of ≤100 nm.	[60]
13	p-Cymene, thymol, and 1,8-cineole from *T. vulgaris* extract	p-Cymene, thymol, and 1,8-cineole are insoluble in water.	Copper chitosan for a nanocomposite hydrogel	Polymeric nanohydrogels were preferred for their biodegradability, biocompatibility, inexhaustibility, non-toxicity, cost-effectiveness, and wide applications.	Thymol nanohydrogel enhances antifungal activity, effective against *Aspergillus flavus*.	The nanohydrogel form of thymol is more water-soluble.	Nanohydrogels exhibit minimal drug-loading efficiency.	[61]
14	Qiai essential oil (QEO)	Essential oils are known for their poor aqueous solubility and high volatility.	Nanohydrogels	The cumulative release rate reached 95% within 35 h.	The nanohydrogel Qiai essential oil enhances antibacterial activity against *S. aureus* and *E. coli* compared to free QEO.	The nanohydrogel Qiai essential oil is more water-soluble.	The encapsulation efficiency and loading capacity of the nanohydrogel Qiai essential oil reaches 80.2% and 6.8%, respectively.	[62]
15	*A. vera* extract	It is a natural polysaccharide with water-soluble characteristics.	Nanohydrogel with chitosan polymers	*Salmonella typhi* was the most sensitive bacterium to all tested nanohydrogel formulations of AV and Ch (except for AV-Ch (3:1 *v*/*w*)). Growth of *Candida albicans* was also inhibited by nanohydrogel AV-Ch (1:1 *v*/*w*) at MIC = 500.	It enhanced wound recovery response by harnessing the potential healing properties of nanohydrogel *Aloe vera*-chitosan.	Nanohydrogel *Aloe vera*-chitosan is highly water-soluble.	Nanohydrogel *Aloe vera*-chitosan exhibits uniform shapes and sizes smaller than 100 nm.	[63]
16	Ginseng extract	Ginseng extract is not soluble in water.	Nanohydrogel with sodium alginate polymer	The diffusion study revealed extended ginseng diffusion from the nanohydrogel for over 20 h.	The ginseng nanohydrogel (NHG) enhanced the potential for wound healing.	The ginseng nanohydrogel (NHG) is highly water-soluble.	The optimized nanoformulation exhibits a particle size of 420.11 ± 5.21 nm, a PDI of 0.424 ± 0.013, a zeta potential of 0.006 ± 0.002 mV, and an encapsulation efficiency of 89.051 ± 0.022%.	[64]
17	Plant polyphenols	Polyphenols are hydrophobic.	Polyphenol zwitterionic nanohydrogel	This nanohydrogel displayed an excellent oil rejection rate (99%) and flux (2100 L·m^−2^·h^−1^·bar^−1^) for up to 30 separation cycles.	Significant improvement in the separation of oily wastewater was achieved.	The polyphenol zwitterionic nanohydrogel is highly water-soluble.	Characterization using Fourier transform infrared spectroscopy and X-ray photoelectron spectroscopy confirms the successful fabrication of the PVDF-Poly(DMA-co-SPP) membrane.	[65]
18	The Mentha plant extract	The Mentha plant extract is insoluble in water.	Nanocomposite hydrogels with k-carrageenan (k-Cr) and chitosan (CS) polymers	These nanocomposite hydrogels showed high toxicity (about 22% at a concentration of 20 μg/mL) against HeLa cells.	They significantly improved antibacterial activity against *S. aureus* (an MIC value of 39.06 μg/mL) and *E. coli* (an MIC value of >19.53).	The nanocomposite hydrogels with k-carrageenan (k-Cr) and chitosan (CS) polymers are highly water-soluble.	These nanocomposite hydrogels exhibit a loading capacity of about 98%.	[66]
19	*Hydnocarpus wightiana* oil	*Hydnocarpus wightiana* oil is hydrophobic and insoluble in water.	Nanohydrogel-based *Hydnocarpus wightiana* oil	This nanohydrogel had the capability for target-specific drug absorption and was biocompatible.	It significantly improved antibacterial and antifungal activity against pathogenic bacteria and fungi, with MIC values ranging from 0.78 to 1.56 μL/mL and effectiveness ranging from 70.29% to 83.62%.	Nanohydrogel-based *Hydnocarpus wightiana* oil is highly water-soluble.	The nanohydrogel exhibits an average droplet size of 103.6 nm with a surface charge of −17.6 mV.	[67]
20	Soy protein isolate	Soy protein isolate is hydrophobic and insoluble in water.	Soy protein isolate nanoparticles (SPI NPs) using Genipin	These nanoparticles showed high self-healing efficiency, reaching 91.6% within 10 h, and exhibited favorable mechanical properties with a tensile strength of 0.89 MPa and strain of 853.2%.	The nanoparticles hydrogel (SPI NPs) by Genipin improved wound healing.	The nanoparticle hydrogel (SPI NPs) by Genipin is highly water-soluble.	An oil-in-water (O/W) Pickering emulsion is formed by SPI NPs encapsulating linseed oil.	[68]
21	*Aloe vera* and *Eucalyptus* essential oil	*Eucalyptus* essential oil is hydrophobic and insoluble in water.	Nanoparticle hydrogel with *Eucalyptus* essential oil, incorporating *E. staigeriana*, loaded with *Aloe vera*, and coated with Dextran Sulfate/Chitosan	The release profile of *E. staigeriana* from the prepared nanoparticles was studied in vitro at a pH of 7.4.	The *E. staggering*-loaded *Aloe vera*-coated *Dextran Sulfate/Chitosan* hydrogel could be an effective dressing material to accelerate wound healing.	The nanoparticle hydrogel *Eucalyptus* essential oil is highly water-soluble.	*Aloe Vera*-coated Dextran Sulfate/Chitosan nanoparticles encapsulated with *E. staigeriana* inhibit bacteria by 47.27%.	[69]
22	*Azadirachta indica* oil	*Azadirachta indica* oil is hydrophobic and insoluble in water.	Nanohydrogel *Azadirachta indica* oil	Chromatography analysis quantified the presence of gallic acid (0.0076 ppm), caffeic acid (0.077 ppm), and syringic acid (0.0129 ppm) in the oil.	Enhanced antimicrobial and anti-inflammatory activities were observed.	Nanohydrogel *Azadirachta indica* oil is highly water-soluble.	The nanohydrogel exhibits a droplet size of 104.1 nm and a zeta potential of −19.3 mV.	[70]
23	Agarose	Agarose is hydrophobic and insoluble in water.	Agarose with selenium nanohydrogel	These nanohydrogels demonstrated good biocompatibility and the ability to enhance photosynthetic efficiency and plant growth.	The nanohydrogels exhibited excellent effects in controlling strawberry gray mold and extending fruit storage time.	Agarose with selenium nanohydrogel is highly water-soluble.	Measurement of fluorescence parameters shows that the maximum photochemical efficiency (Fv/Fm) of plant leaves in the inoculated group (*B. cinerea*) is 0.58. In contrast, the Fv/Fm value of the nanohydrogel group exceeds 0.8.	[71]
24	Guar gum	Guar gum is hydrophobic and insoluble in water.	Guar gum-crosslinked-Soya lecithin nanohydrogel	It achieved a maximum adsorption capacity of 59.205 mg/g.	The improved spontaneous and endothermic adsorption process was observed.	Guar gum-crosslinked lecithin nanohydrogel is highly water-soluble.	It is observed with a 20 mg GG-crosslinked-SY NHS and a 25 ppm thiophanate methyl solution concentration, as calculated from the Langmuir isotherm.	[72]
25	Alginate (Alg)	Alginate is insoluble in water.	Injectable nanohydrogel paste based on natural alginate (Alg) derived from brown sea algae as a polysaccharide polymer	The nanohydrogel paste reduced healing time and fully restored well-mature bone tissue, similar to natural bone.	Injectable nanohydrogels Alginate/n-Hydroxyapatite (Alg/n-HA) exhibited appreciable biodegradability and bioactivity.	Nanohydrogel Alginate/n-Hydroxyapatite (Alg/n-HA) is highly water-soluble.	The viscosity and mechanical properties of the paste are investigated, as well as in-vitro studies related to water absorption and biodegradability in PBS.	[73]

**Table 2:** Comparison of IC_50_ phytochemical compounds with nanohydrogels [24,34].

Phytochemical Compounds	Method	IC_50_ Extract/Fraction/Isolate		IC_50_ Nanohydrogel		Ref.
Curcumin	MTT Test	In 4T1 cells, it is 2 µg/mL.	In MiaPaCa-2 cells, it is 9 µg/mL.	In 4T1 cells, it is 5 µg/mL.	In MiaPaCa-2 cells, it is 18 μg/mL.	[46]
Apigenin	MTT Test and Trypan Blue Staining	K562 cells increased by 20 mmol after 48 h.	48 h = 20 ± 1.66 µmol/mL; 48 h = 20 ± 0.82 µmol/mL.	K562 cells increased by 10 mmol after 48 h.	48 h = 10 ± 0.53 µmol/mL; 48 h = 10 ± 1.98 µmol/mL.	[30]

## Data Availability

Data sharing is not applicable.
